# Anemia in Children with Congenital Heart Disease: A Finding from Low-Resource Setting Hospitals

**DOI:** 10.1155/2024/8095150

**Published:** 2024-04-12

**Authors:** Henok Kumsa, Rediet Woldesenbet, Feven Mulugeta, Rajalakshmi Murugan, Tamirat Moges

**Affiliations:** ^1^School of Midwifery, College of Health Science, Woldia University, Woldia, Ethiopia; ^2^School of Nursing and Midwifery, College of Health Science, Addis Ababa University, Addis Ababa, Ethiopia; ^3^School of Medicine, College of Health Science, Addis Ababa University, Addis Ababa, Ethiopia

## Abstract

**Introduction:**

Congenital heart disease (CHD) is the most common birth defect. Anemia is the prevailing manifestation of micronutrient deficiency. It has been demonstrated that anemia in children increases morbidity and has a negative impact on psychomotor development. Despite its negative consequences, which have been documented for a long time in clinical practice, the issue does not gain sufficient attention in developing countries, specifically in children with CHD. Thus, this study is aimes to assess the prevalence of anemia and the factors associated in children with CHD.

**Methods:**

Institutional-based cross-sectional study was conducted on CHD children at selected governmental hospitals in Addis Ababa, Ethiopia, from February to March 2021. During this period, 373 children with acyanotic and cyanotic heart disease between 0 months and 15 years of age were included in this study. All children were assessed using structured questionnaires and anthropometric measurements. Recent hemoglobin results that are avaliable in the medical charts of children were used to diagnose anemia. The data were analyzed using SPSS version 25.

**Results:**

From randomly included 373 children with CHD, 298 (79.9%) had acyanotic congenital heart disease (ACHD) and 75 (20.1%) had cyanotic congenital heart disease (CCHD). Twenty-five (33.3%) CCHD and 192 (64.4%) ACHD cases of children were malnourished. The most common type of CCHD and ACHD defects were ventricular septal defects and tetralogy of fallout, respectively. Overall, the prevalence of anemia in ACHD and CCHD was 24.5% and 72%, respectively. In children with ACHD, the frequency of anemia was reported to be significantly higher in the malnourished group than in the well-nourished.

**Conclusions:**

A high prevalence of anemia is observed in children with CHD. This study highly suggests further evaluation to determine the frequency and complications of blood indices and other hematological impairments in ACHD, CCHD, and children with both problems. Moreover, the findings of this study on illness profiles in children with CHD prompt further research into the cellular and molecular mechanisms underlying immune system dysfunction.

## 1. Introduction

Congenital heart disease is defined as a defect in the structure of the heart walls and vessels that present at birth, which accounts for the incidence rate of 8/1000 among live births [1]. Congenital heart disease is divided based on its relationship with oxygen as acyanotic and cyanotic congenital heart defect. Additionally, it is classified based on complexity of separation of the defect into three groups: simple, moderately complex, and complex defect. The simple group consists of ventricular septal defect, atrial septal defect, pulmonary stenosis, and patent ductus arteriosus. Moderately complex consists of tetralogy of Fallot, aortic stenosis, and pulmonary stenosis. Complex defects consist of double outlet right ventricle, tricuspid atresia, pulmonary atresia, congenitally corrected transposition of great arteries, and atrioventricular septal defect [2].

Anemia is defined as a decrement in number of red blood cells circulating in the cardiovascular system compared to the normal levels and commonly measured using hemoglobin or hematocrit count [3]. Anemia reduces physical performance in adults and especially to exert negative effect on the psychomotor development in children and adolescents [4, 5]. Iron deficiency with or without anemia during infancy period is associated with increased behavioral and social problems in youth [5]. Importantly, anemia in children with congenital heart disease showed to be a major risk factor that increases morbidity and mortality [6].

Children with untreated congenital heart disease, particularly those with tetralogy of Fallot, often experience a backflow of blood, leading to chronic desaturation. This may result in an increased erythrocyte count and elevated blood viscosity, which contributes to the depletion of iron stores [7]. Even though polycythemia is associated with children who have cyanotic CHD, mostly both acyanotic and cyanotic children have the same high risk of having iron store depletion and iron deficiency anemia [8].

In developed nations, CHD is currently treated in the early stages with optimal nutritional support, while a large proportion of children with complicated congenital cardiac abnormalities is untreated in low-income countries like Ethiopia. On top of it, the high prevalence of malnutrition is still a public health concern in children [9, 10]. Thus, this study is aimes assess the prevalence of anemia in children with congenital heart defects using hemoglobin levels and identifying associated factors.

## 2. Methods

### 2.1. Study Area

This study was conducted in governmental hospitals and cardiac center of Addis Ababa, Ethiopia. Addis Ababa is the capital city of Ethiopia and seat of African Union and the United Nations World Economic Commission for Africa. It covers an area of 527 square kilometers and has 11 subcities with a population of 3,384,569 according to the 2007 census [11]. The city has subtropical highland climate. Addis Ababa has 13 governmental public hospitals, including one university hospital, six federal hospitals, and six regional hospitals. Three hospitals (Black Lion Hospital, St. Peter Specialized Hospital, and Yekatit 12 Hospital) and Ethiopian cardiac center were chosen for the study because they have an outpatient department for children with cardiac cases.

### 2.2. Study Design, Population, and Period

It was institution-based cross-sectional study on different types of congenital heart defects at selected governmental hospitals in Addis Ababa, Ethiopia. The study participants were children aged less than 15 years and had follow-up in respective hospitals. The study was conducted from February 27 to March 25, 2021.

### 2.3. Inclusion and Exclusion Criteria

Children who have confirmed CHD age less than 15 years at the time of study and come to cardiac OPD for their follow-up were included in the study, whereas children who are on iron supplements and/or have had blood transfusions in the past three months were excluded.

### 2.4. Sample and Sampling Procedure

The study included 373 eligible children in the selected hospitals. The sample size was calculated using single proportion formula. It is determined by using the prevalence of CHD 6.5% among admitted children study conducted at Hawassa University, comprehensive specialized hospital Ethiopia, and margin of error 0.25% [12]. Three of the hospitals (Tikur Anbessa Hospital, St. Peter Specialized Hospital, and Yekatit 12 Hospital) and an Ethiopian cardiac center were selected for conducting the research. Children who came for follow-up to cardiac center hospitals prior to 1 month of the data collection were counted to determine the propertional allocation. Study participants were selected by consecutive sampling technique until the final sample size was attained.

### 2.5. Study Variables

#### 2.5.1. Dependent Variable: Anemia

#### 2.5.2. Independent Variable

Sociodemographic characteristics were age, sex, birth order, parental education, religion, parental occupation, and residency. Child medical conditions were child illness, type of CHD (cyanotic and acyanotic), pulmonary hypertension, heart failure, and corrective surgery. Dietary factors were complementary feeding, feeding practice, and feeding problems.

### 2.6. Operational and Standard Definition

- Children for this study age group are taken as child age below 15 years
- Congenital heart defect: major or minor congenital anomalies defined as anatomical structural and functional defect present at birth which was confirmed by pediatricians with echocardiography
- Adequate (good) dietary diversity: when children have 4 or more food group dietary diversity per day
- Inadequate (poor) dietary diversity: when children have less than 4 food group dietary diversity per day
- Malnourished: children who scored <-2SD in either one of weight-for-height *Z*-score (wasting), weight-for-age *Z*-score (underweight), and height-for-age *Z*-score (stunting). Additionally, body mass index *Z*-score for children above 10 years of age [13]
- Anemia: hemoglobin less than 12 g/dl for children with acyanotic congenital heart disease and hemoglobin less than 15 g/dl for children with cyanotic congenital heart disease [14]
- Categories of anemia using WHO classfication method as presented in Table 1 [15]

### 2.7. Data Collection Method

Before conducting data collection, structured questionnaire was prepared after reviewing the literature's and pretest was done on 5% of the study participants. The data was collected by face-to-face interview, reviewing medical charts to collect findings related with diagnosis of CHD and last hemoglobin level. Moreover, to assess malnutrition status of the child, anthropometric data of the children were measured during data collection. The data collection instrument consists of four parts: part one: anthropometric measurement; part two: sociodemographic characteristics; part three: dietary history; and part four: child medical conditions.

### 2.8. Data Processing and Analysis

After editing and sorting data were entered into EpiData version 4.6 and analyzed using SPSS version 25. Independent sample *t*-test was used to compare between two independent groups, and *P* value < 0.05 was considered significant.

### 2.9. Ethical Consideration

Ethical clearance was obtained from the institutional review board of Addis Ababa Health Bureau. Official letter of permission was written from the department to the respective hospitals. Written consent was obtained from each respondent (parents and guardians) after explaining the purpose and procedure of the study. Personal identifying information was not included in the instrument used for data collection. Additionally, the authors had no access to information that could identify individual participants during or after data collection.

## 3. Result

### 3.1. Sociodemographic Characteristic Study Participants

The study was conducted on 373 children, and among them, 298 (79.9%) have acyanotic congenital heart disease (ACHD) and 75 (20.1%) were diagnosed for cyanotic CHD. The median age of children with ACHD was 45 months and range of 171 months. One hundred sixty-nine children with ACHD cases were below the age of 59 months, and 251 (84.2%) ACHD children lived in family size of four and above. Nearly one-third (33.2%) of the ACHD children's mothers have a secondary education, and 274 (91.9%) children were urban dwellers. Moreover, the fathers of children with CCHD cases (28 (37.3%)) were employed in governmental or nongovernmental institutions (Table 2).

### 3.2. Dietary History and Nutritional Status of CHD Children

Children less than two years old were assessed for breastfeeding status and nutritional status by using structured checklist and anthropometric measurement. Children above 60 months old assessed their dietary history by using Ethiopian demographic health survey checklist format. The mothers of the one hundred sixty-six children have obtained information about nutrition. Nearly three-fourths of children below 59 months with ACHD cases have obtained colostrum. Moreover, 144 (48.3%) and 111 (37.2%) children with ACHD have feeding difficulty and history of illness in the last two weeks, respectively. Using the dietary diversity evaluation system, when children have four or more food group dietary diversity per day, it is considered “good dietary” diversity; if it is below four, it is considered inadequate or “poor dietary.” Based on this evaluation instrument, 45 (64.3%) CCHD cases in children were considered or categorized as having a good dietary history. Lastly, 192 (64.4%) ACHD children were malnourished (Table 3).

### 3.3. Types of Congenital Heart Disease and Comorbidity

Table 4 shows the types of congenital heart disease and comorbidity observed in the main study groups. The most common type of ACHD was ventricular septal defect, followed by atrial septal defect and atrioventricular septal defect. The most common cyanotic congenital heart disease was tetralogy of Fallot. Twenty-two (7.4%) of 298 children with ACHD have been diagnosed with heart failure. Moreover, forty (13.42%) children with ACHD were operated for corrective surgery.

### 3.4. Prevalence of Anemia Using Hemoglobin for Acyanotic and Cyanotic CHD

In this study, among 298 children with ACHD, 73 (24.5%) had hemoglobin less than 12 mg/dl or were anemic, whereas in children with CCHD, 54 (72%) of the 75 CCHD children had hemoglobin levels below 15 mg/dl or were considered anemic. In addition, according to the WHO's classification of anemia, 11 out of 108 children in the 60–132-month-age range had moderate anemia (Table 5). Moreover, the mean hemoglobin in CCHD cases was 14.07 gm/dl with a S.D. of 3.35, whereas children with ACHD had a lower mean hemoglobin level of 13.04 gm/dl with a S.D. of 2.39.

### 3.5. Comparison of Anemia among Children with CHD

Independent sample *t*-test was used to compare between two independent groups of normally distributed variables, and *P* value < 0.05 was considered significant. The children have been divided into two groups regarding hemoglobin count: 73 (24.5%) children with anemia and 225 (75.5%) children with a normal hemoglobin count or above 12 mg/dl. There was no significant difference between two groups of anemic and nonanemic children regarding sex (*P* value = 0.841) and family size (*P* value = 0.966). Out of 73 anemic children, 54 were found to have malnutrition (*P* value = 0.023). In other words, the frequency of anemia was reported significantly higher in the malnourished group than in the well-nourished. Likewise, children with CCHD cases complicated with pulmonary hypertension showed significant association with anemia (*P* value = 0.019). Furthermore, acyanotic and cyanotic heart disease cases that were assessed between two groups of anemic and nonanemic children are presented in Tables 6 and 7.

## 4. Discussion

Different types of cardiac malformation can affect blood index levels like hemoglobin, white blood cell, and neutrophil count in varying degrees [16]. Our study was conducted to evaluate the status of anemia using only hemoglobin levels. The prevalence of anemia based on hemoglobin levels lower than 12 mg/dl was 24.5% in children with ACHD, whereas it was 72% in children with CCHD for hemoglobin levels lower than 15 mg/dl. There is a paucity of literature to discuss anemia among children with CCHD and ACHD using hemoglobin levels.

The study conducted by Amoozga et al. showed the prevalence of anemia using an Hgb level below 12 mg/dl, among 60 patients with ACHD was 50.7%, while in the cyanotic group about 75.9% were had Hgb < 15 gm/dl; the result showed higher difference in ACHD between the current study and our finding [14]. This might be due to the differences in the sample size, study period, and sociodemographic characteristics.

Addtionally, this study revealed that high prevalence of anemia in children with CCHD compared to the study conducted by Banu Onur et al. which demonstrated that not more than one-third of children with CCHD had iron deficiency anemia [17]. In another study done by Olcay et al. and Soni et al., the prevalence of iron deficiency anemia was found to be 52.2% and 56.6, respectively [18, 19]. Anemia was overdiagnosed in our study since it was diagnosed based solely on hemoglobin levels; this could explain the difference with the mentioned studies. Additionally, nearly half of the children had mixed CHD problems; therefore, this might exaggerate the prevalence of anemia in our finding. Relatively, our finding is equivalent or relatively similar finding with the studies by Mohammadi et al. and Gaiha et al. with a prevalence of 18.18% and 19.3% [16, 20]. However, iron deficiency anemia is reported to be common in individuals with CCHD even in the presence of high hematocrit levels. This explanation strengthen by the study conducted in India showed that hemoglobin and hematocrit levels and RBC count were paradoxically higher in the CCHD as compared to the healthy controls [21]. Regarding the ACHD cases, the prevalence of anemia in our study was equivalent to that found in studies conducted by Mohammadi et al. and Gaiha et al. [16, 20].

The mean hemoglobin in CCHD cases was 14.07 gm/dl with a S.D. of 3.35, which was a higher mean hemoglobin level than children with ACHD (mean 13.04 gm/dl with a SD of 2.39). Similarly, high level of mean hemoglobin reported from the studies conducted in Turkey, Iran, and Nigeria found mean hemoglobin of CCHD cases was 14.8 gm/dl, 16.0 gm/dl, and 17.0 gm%, respectively [14, 17, 18]. This might be explained by renal hypoxia in CCHD cases, which triggers the release of erythropoietin, a hormone that stimulates the bone marrow to produce more red blood cells. This increase in red blood cell production leads to higher red cell mass in the body [22, 23].

In our study, a statistically significant association has been seen in between anemia and malnutration in children with ACHD. However, there was no statistically significant difference in the occurrence of anemia with the types of ACHD, age, and family size. Similar finding was revealed in study conducted in Kenya [24]. In case of children with CCHD, statistically significant association has been seen between anemia and pulmonary hypertension.

The current study's limitations are based on its small sample size. As a result, it may not be sufficiently powered to draw major statistical conclusions. Another problem is that the study population also included infants under the age of six months, which could raise the mean values of the hematological parameters.

## 5. Conclusions

There is a high prevalence of anemia in children with ACHD and CCHD. Furthermore, CCHD children, in particular patients with pulmonary hypertension, should be considered a group at higher risk of anemia. This study emphasizes the importance of proper nutritional rehabilitation for these children, with a focus on iron supplementation. Besides, this study highly suggests further evaluation to determine the blood indices and other hematological impairments in ACHD, CCHD, and children with both cases. Finally, the high level of illness observed in children with CHD warrants further research to explore the cellular and molecular basis of immune system dysfunction, with the aim of improving the quality of life for these children.

## Figures and Tables

**Table 1:** Categories of anemia using WHO classification method.

Age in month	Classification of anemia			
	Severe	Moderate	Mild	Normal value
6-59	<6.9 gm/dl	7-9.9 gm/dl	10-10.9 gm/dl	>10.9 gm/dl
60-132	<7.9 gm/dl	8-10.9 gm/dl	11-11.4 gm/dl	>11.4 gm/dl
Above 132	<7.9 gm/dl	8-10.9 gm/dl	11-11.9 gm/dl	>11.9 gm/dl

**Table 2:** Sociodemographic characteristics of children with congenital heart disease in selected governmental hospitals and cardiac center, Addis Ababa, Ethiopia (2021).

Variables	ACHD		CCHD	
	Freq.	Perc. (%)	Freq.	Perc. (%)
Age of a child				
0-59 months	169	56.7	46	61.33
60–120 months	95	31.9	22	29.33
>120 months	34	11.4	7	9.33
Sex of a child				
Male	144	48.3	39	52
Female	154	51.7	36	48
Residence				
Rural	24	8.1	7	9.4
Urban	274	91.9	68	90.6
Family size				
0-3	47	15.8	58	77.3
4 and above	251	84.2	17	22.7
Birth order				
First	106	35.6	33	44
Second and third	144	48.3	27	36
Fourth and above	48	16.1	15	20
Educational status mother				
No formal education	44	14.8	12	16
Primary education	80	26.8	20	26.7
Secondary education	99	33.2	27	36
College and above	75	25.2	16	21.3
Occupational status mother				
Housewife	154	51.7	33	44
Non/governmental employee	64	21.5	17	22.7
Merchant	35	11.5	9	13.3
Farmer	18	6	10	12
Daily labor	20	6.7	6	8
Self-employed	7	2.3	—	—
Educational status (father)				
Unable to write and read	30	10.1	9	12
Primary education	67	22.5	11	14.7
Secondary education	89	29.9	26	34.7
College and above	102	34.2	24	32
Child not lived his/her father	10	3.4	5	6.7
Occupational status of father				
Non/governmental employee	138	46.3	28	37.3
Merchant	51	17.1	9	12
Farmer	20	6.7	11	14.7
Daily labor	30	10.1	6	8
Self employed	42	14	7	9.3
Unemployed	7	2.3	9	12
Child not lived his/her father	10	3.4	5	6.7

**Table 3:** Nutritional status, dietary, and health history of children with congenital heart disease in selected governmental hospitals and cardiac center, Addis Ababa, Ethiopia (2021).

Variables	ACHD		CCHD	
	Freq. (298)	Perc. (%)	Freq. (75)	Perc. (%)
Having information about nutrition				
Yes	166	55.7	49	65.3
No	132	44.3	26	34.7
Is the child under 60 months				
Yes	104	34.9	28	37.3
No	194	65.1	47	63.7
When start breastfeeding				
With one hour	64	61.5	19	67.9
After one hour	40	38.5	9	32.1
Does your children get colostrum				
Yes	77	74	24	85.7
No	27	26	4	14.3
Do you still breastfeed				
Yes	77	74	21	75
No	27	26	7	25
Frequency of breastfeeding per day				
<8 times	67	84.1	2	9.5
8 and above times	10	14.9	19	90.5
Do you start bottle-feeding				
Yes	71	69.2	17	60.7
No	33	31.8	11	39.3
Have you started complementary feeding				
Yes	73	70.2	23	82.1
No	31	29.8	5	17.9
At what age have you started complementary feeding				
<6 month	61	83.6	21	91.3
6 month and above	12	16.4	2	8.7
Does the child has feeding difficulty				
Yes	144	51.7	35	43.3
No	154	48.3	40	46.7
Dietary history for age above 50 months				
Good	184	68.9	45	64.3
Poor	83	31.1	25	35.7
Nutritional status				
Well nourished	106	35.6	50	66.7
Malnutrition	192	64.4	25	33.3
Does your child got sick in the past two weeks				
Yes	111	62.8	19	23.3
No	187	37.2	56	76.7

**Table 4:** Types of congenital heart disease and comorbidities of children with congenital heart disease in selected governmental hospitals and cardiac center of Addis Ababa, Ethiopia (2021).

Acyanotic		ACHD and CCHD (both)	Cyanotic	
Types of congenital heart disease	Frequency (298)	Frequency (31)	Types of congenital heart disease	Freq. (75)
Ventricular septal defect	123	14	Tetralogy of fallout	32
Atrial septal defect	73	8	Transposition of great arteries	12
Atrioventricular septal defect	32	4	Total anomalous pulmonary venous return	9
Patent ductus arteriosus	101	11	Pulmonary atresia	9
Coarctation of aorta	17	1	Hypoplastic left heart syndrome	1
Pulmonary stenosis	23	7	Truncus arteriosus	6
Aortic valve stenosis	3	—	Double outlet right ventricle	10
Patent foramen ovale	14	—	—	
Pulmonary hypertension	114	—	—	24
Heart failure	22	—	—	4
Corrective surgery conducted	40	—	—	15

**Table 5:** Prevalence of anemia in congenital heart disease children in selected governmental hospitals and cardiac center of Addis Ababa, Ethiopia (2021).

Age in month	Anemia classification for ACHD based on Hgb level				Prevalence freq. (%)
	Severe freq.	Moderate freq.	Mild freq.	Normal value freq.	
6-59 (138 children)	0	5	15	118	20 (14.49%)
60-132 (108 children)	0	11	7	90	18 (16.66%)
Above 132 (21 children)	0	3	2	16	5 (23.8%)
Overall anemia for children with ACHD (298) (hemoglobin level less than 12 mg/dl)					73 (24.5%)
Over all anemia for children with CCHD (75) (hemoglobin level less than 15 mg/dl)					54 (72%)

**Table 6:** Clinical data of ACHD children with anemia compared to normal hemoglobin level in selected governmental hospitals and cardiac center of Addis Ababa, Ethiopia (2021).

Variables	Hgb < 12 g/dl	Hgb ≥ 12 g/dl	Total	*P* value
Sex				
Male	35	109	144	
Female	38	116	154	0.841
Family size				
0-3	12	35	251	
4 and above	61	190	47	0.966
Ventricular septal defect	22	101	123	0.292
Atrial septal defect	11	62	73	0.677
Atrioventricular septal defect	5	27	32	0.060
Patent ductus arteriosus	10	91	101	0.162
Coarctation of aorta	3	14	17	0.602
Pulmonary stenosis	3	20	23	0.380
Aortic valve stenosis	1	2	3	0.539
Patent foramen ovale	3	11	14	0.629
Pulmonary hypertension	20	94	114	0.583
Malnutrition				
Well nourished	19	87	106	
Malnutrition	54	138	192	0.023^∗∗^

Hgb: hemoglobin. ^∗∗^Significantly associated.

**Table 7:** Clinical data of CCHD children with anemia compared to normal hemoglobin level in selected governmental hospitals and cardiac center of Addis Ababa, Ethiopia (2021).

Variables	Hgb < 15 mg/dl	Hgb ≥ 15 mg/dl	Total	*P* value
Sex				
Male	28	11	39	
Female	26	10	36	0.949
Family size				
0-3	42	9	51	
4 and above	12	5	17	0.848
Tetralogy of Fallot	23	9	32	0.860
Transposition of great arteries	9	3	12	0.677
Pulmonary hypertension	15	9	24	0.019^∗∗^
Nutrition status				
Well nourished	38	12	50	
Malnutrition	16	9	25	0.796

Hgb: hemoglobin. ^∗∗^Statistically significant.

## Data Availability

The datasets used in this study are available from the corresponding author upon reasonable request.

## Abbreviations

ACHD: Acyanotic congenital heart defect/disease
CCHD: Cyanotic congenital heart defect/disease
CHD: Congenital heart defect/disease
MUAC: Midupper arm circumference
SD: Standard deviation
WHO: World Health Organization.
